# Supplementary material for: The Root Nodule Microbiome of Cultivated and Wild Halophytic Legumes Showed Similar Diversity but Distinct Community Structure in Yellow River Delta Saline Soils
Source: Microorganisms. 2020 Feb 3;8(2):207. doi: 10.3390/microorganisms8020207 (PMC7074777; doi:10.3390/microorganisms8020207)
Supplement: Supplementary file 1 [file microorganisms-08-00207-s001.pdf]

microorganisms

MDPI

Supplementary Materials

The root nodule microbiome of cultivated and wild halophytic legumes showed similar diversity but distinct community structure in Yellow River Delta saline soils

Yanfen Zheng [1†], Jing Liang [1,2†], Dong-Lin Zhao [1],Chen Meng [1,2], Zong-Chang Xu [1,2], Zhi-Hong Xie[3,*], Cheng-Sheng Zhang [1,2,*]

[1] Marine Agriculture Research Center, Tobacco Research Institute of Chinese Academy of Agricultural Sciences, Qingdao, 266101, China; zhaodonglin@caas.cn (D.-L.Z.); zhengyanfen@caas.cn (Y.Z.); 13739089026@163.com (J.L.); mengchen01@caas.cn (C.M.); xuzongchang@caas.cn (Z.-C.X.); zhchengsheng@126.com (C.-S.Z.)

[2] Special Crops Research Center of Chinese Academy of Agricultural Sciences, Qingdao, 266101, China; 13739089026@163.com (J.L.); mengchen01@caas.cn (C.M.); xuzongchang@caas.cn (Z.-C.X.); zhchengsheng@126.com (C.-S.Z.)

[3] Yantai Institute of Coastal Zone Research, Chinese Academy of Sciences, Yantai, 264003, China; zhxie@yic.ac.cn

†These authors contributed equally to this work

*Correspondence: zhchengsheng@126.com; Tel.: +86-0532-8870-2115; zhxie@yic.ac.cn

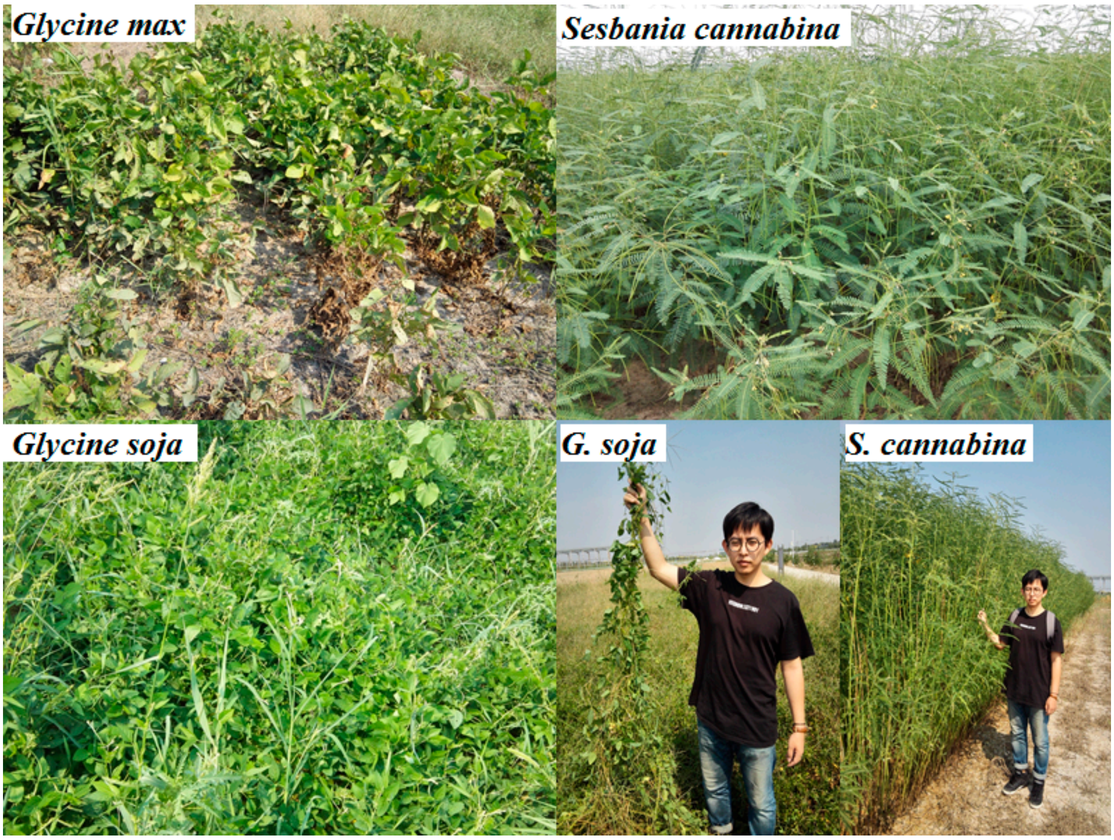

Figure S1: Investigations of the growth of *S. cannabina*, *G. soja*, and *G. max* in the field

Table S1. 16S rRNA read counts and number of identifiable units at different taxonomical levels

Sample	Raw_Tags	Final_Tags	OTUs	Phylum	Genus	Species
Gs1	9024	2449	181	1	7	35
Gs2	6252	1433	210	3	18	53
Gs3	7478	2213	184	1	6	32
Sc1	5393	1524	157	1	2	11
Sc2	3869	1086	150	1	2	11
Sc3	5096	1365	156	1	2	11
Gm1	11180	2707	264	2	9	31
Gm2	4918	1015	207	2	12	44
Gm3	5813	1342	179	2	13	43

Table S2. Operational taxonomic units common to 75% of samples

OTU ID	Genus/Species	Gm1	Gm2	Gm3	Gs1	Gs2	Gs3	Sc1	Sc2	Sc3
denovo1901	*Ensifer*	9	1	1	0	19	0	45	28	33
denovo8435	*Ensifer*	4	1	0	0	10	1	13	7	8
denovo10033	*Ensifer*	32	13	1	0	40	1	103	86	104
denovo11873	*Ensifer/unidentified*	14	5	2	6	10	5	2	0	0
denovo12569	*Ensifer/unidentified*	29	13	4	0	45	3	67	29	29
denovo12913	*Ensife/unidentified*	7	2	1	0	10	0	1	2	2
denovo13709	*Ensifer*	7	2	0	0	6	1	10	9	11
denovo16547	*Ensifer*	43	13	0	0	97	3	121	100	122
denovo20293	*Ensifer/unidentified*	36	11	1	0	36	2	79	49	97
denovo20381	*Ensifer/unidentified*	45	15	23	82	9	66	0	0	1
denovo25011	*Ensifer*	22	8	0	0	39	1	84	59	101
denovo26571	*Ensifer*	2	1	0	0	1	1	4	7	6
denovo27243	*Ensifer*	6	3	1	0	11	0	9	10	8
denovo29807	*Ensifer/unidentified*	16	7	8	6	9	11	2	2	2
denovo33526	*Ensifer/unidentified*	517	246	399	680	236	637	9	5	2
denovo35463	*Ensifer/unidentified*	27	6	1	2	42	3	0	1	5
